# Novel insights into the interplay between m6A modification and programmed cell death in cancer

**DOI:** 10.7150/ijbs.81000

**Published:** 2023-03-13

**Authors:** Jinhao Chen, Mujie Ye, Jianan Bai, Chunhua Hu, Feiyu Lu, Danyang Gu, Ping Yu, Qiyun Tang

**Affiliations:** Department of Geriatric Gastroenterology, Neuroendocrine Tumor Center, Jiangsu Province Hospital, The First Affiliated Hospital of Nanjing Medical University, Institute of Neuroendocrine Tumor, Nanjing Medical University, Nanjing, China.

**Keywords:** N6-methyladenosine, cancer, programmed cell death, apoptosis, autophagy, pyroptosis, ferroptosis, necroptosis

## Abstract

N6-methyladenosine (m6A) methylation, the most prevalent and abundant RNA modification in eukaryotes, has recently become a hot research topic. Several studies have indicated that m6A modification is dysregulated during the progression of multiple diseases, especially in cancer development. Programmed cell death (PCD) is an active and orderly method of cell death in the development of organisms, including apoptosis, autophagy, pyroptosis, ferroptosis, and necroptosis. As the study of PCD has become increasingly profound, accumulating evidence has revealed the mutual regulation of m6A modification and PCD, and their interaction can further influence the sensitivity of cancer treatment. In this review, we summarize the recent advances in m6A modification and PCD in terms of their interplay and potential mechanisms, as well as cancer therapeutic resistance. Our study provides promising insights and future directions for the examination and treatment of cancers.

## Introduction

Studies have revealed that more than 160 chemical modifications occur in RNA molecules, and m6A modification is the sixth N atom of adenine to be methylated 1. It is the most common post-transcriptional modification and exerts a variety of essential biological functions on mRNA and ncRNA 2, 3. M6A modification sites are often located in stop codons and the 3'-UTR region with a typical consensus sequence RRACH (R = G or A and H = A, C, or U) 4, and are regulated by writers, erasers, and readers. Studies have indicated that m6A modification is associated with many physiological processes, including the alternative splicing of pre-mRNAs, mRNA degradation, mRNA stabilization, miRNA processing, and cap-independent translation 5. Additionally, m6A plays an indispensable role in cancer and other diseases 6,7.

Programmed cell death (PCD) is an active and orderly process of cell death, including apoptosis, autophagy, ferroptosis, pyroptosis, and necroptosis 8. To balance the homeostasis of the internal environment, PCD can clear and maintain abnormal cells. In addition, aberrant PCD, which has been extensively manipulated to affect the development of cancer and other diverse diseases, has received increasing attention 9. Since the regulation between m6A and apoptosis was first elaborated, emerging studies have focused on abnormal m6A levels as key regulators of PCD. In this review, we introduce the complex role of m6A and the latest progress regarding the connection between m6A modifications and PCD in diverse diseases. Additionally, the future clinical applications of m6A-modified PCD in chemotherapies and precision medicine are also discussed.

### M6A regulators

**Figure 1:**
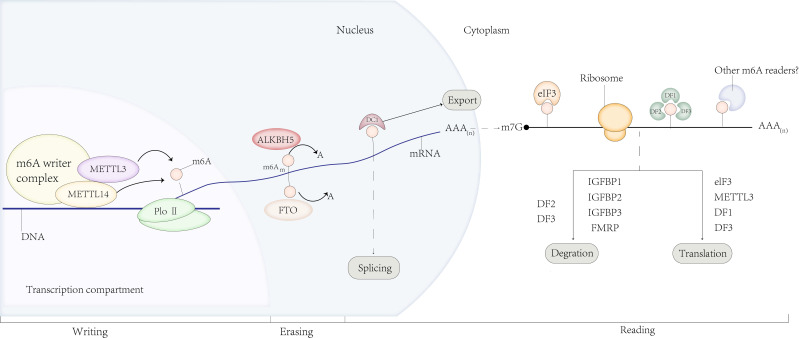
M6A regulators. M6A is deposited by writers, removed by erasers, and recognized by readers. Regulatory functions of m 6 A modification in RNA splicing, processing, translation and degradation.

M6A modification is a dynamic and reversible progression that is modulated by methyltransferases and demethylases 10(Fig.1). Regarding the M6A writers, such as methyltransferase-like protein 3/14 (METTL3/14), Wilms tumor-associated protein (WTAP), and vir-like m6A methyltransferase associated (VIRMA) 11 are the key proteins responsible for catalyzing m6A methylation on RNA 12. METTL3 and METTL14 are the catalytic cores of MTCs 13. Methyltransferase-like protein 3 (METTL3), an S-adenosylmethionine (SAM)-binding protein, catalyzes the transfer of methyl groups in SAM 14. Methyltransferase-like protein 14 (METTL14), another active component of MTC, is a support enzyme devoid of catalytic activity and can form a stable complex with METTL3 to stabilize the structure of MTC 15. Other subunits, such as Wilms tumor1-associated protein (WTAP) 16 and RNA binding motif protein 15 (RBM15) 17, can attract MTCs to specific regions of RNA to induce methylation. METTL3, METTL5 18, and METTL16 19, the newly discovered independent RNA methylases, as catalytic subunits, can also catalyze m6A modification via methyltransferase domains. Zinc finger CCCH domain-containing protein 13 (ZC3H13) enhances the MTC catalytic function by interacting with WTAP 20. Conversely, m6A erasers, such as fat mass and obesity-associated protein (FTO) and ALKB homolog 5 (ALKHB5), perform the function of demethylating m6A modified bases 21,22. ALKBH3, a newly discovered demethylase, can also participate in this process via a similar mechanism 23. Moreover, m6A readers, m6A binding proteins recognize m6A modifications, including YTH domain-containing family protein (YTHDF1-3 and YTHDC1-2) 24, 25, 26, 27, heterogeneous nuclear ribonucleoprotein (HNRNP) protein families 28, eukaryotic translation initiation factor 3 (eIF3) 29, and insulin-like growth factor-2 mRNA-binding proteins 1/2/3 (IGF2BP1/2/3) 30, 31, 32, and identify and bind to m6A marks directly to regulate downstream mRNA translation, decay, and stability.

### M6A modifications in RNA metabolism

As noted above, m6A methylation elicits a wide range of efforts on mRNA processing, splicing, translation, and decay by m6A writer-complex component and m6A reader, as well as erasers that affect RNA metabolism to determine RNA fate and function 5. The importance of m6A as a post-transcriptional modification has been recognized, but the evolution, function and regulation of individual m6A sites remain largely unknown. M6A modification sites are broadly found in stop codons and the 3'-UTR regions with the consensus sequence RRACH (in which R represents A or G, and H represents A, C or U) 4. M6A methylation sites may appear with different functional consequences depending on their location. Concretely speaking, some research revealed that transcripts with m6A in the 5′UTR and/or CDS were related to energy metabolism, mitochondrial function and intracellular signals, while methylation of transcripts in the 3′UTR mostly code for proteins involved in pathways linked to more specific metabolic processes such as 'acetyl-CoA or glycerol biosynthesis' and 'positive regulation of protein dephosphorylation' 33.

RNA metabolism to be more specific. For m6A modification in RNA processing, METTL3 labeled pri-miRNAs for recognition and processing by DGCR8 and promotes the initiation of miRNA biogenesis 34. Depletion of HNRNPA2B1 reduced the processing of primary microRNA-106b to enhance NSCLC cell growth 35. For m6A modification in RNA splicing, m6A modification prevents the essential splicing factor U2AF35 from recognizing the 3' splice site to inhibit RNA splicing 36. FTO regulates nuclear mRNA alternative splicing by binding with SRSF2 37. YTHDC1 enhances an oncogenic RNA splicing of tumor suppressor RBM4 38. For m6A modification in RNA degradation, YTHDF2 changes the localization of bound mRNA from the translatable pool to mRNA decay sites to regulate mRNA degradation 39. In colorectal cancer, YTHDF2 can also upregulate the mRNA stability of lncRNA STEAP3-AS1 to affect the development of cancer 40. METTL3 delays SOCS2 mRNA degradation to regulate liver cancer progression 41. For m6A modification in RNA translation, m6A in 3 'UTRs was found to improve translation efficiency by binding to YTHDF130^42^, while m6A in 5' UTRs was reported to promote cap-independent translation under heat shock stress 43. M6A modifications can be found in mRNA and noncoding RNA (ncRNAs) to regulate gene expression in its 5′ or 3′ UTR. M6A modification not only drives translation on mRNA but also unexpected works on some ncRNAs 44, 45.

## The double sword of m6A in human cancers

High-profile RNA epigenetic modification N6-methyladenosine (m6A), as a double-edged sword for cancer, can either promote or inhibit the occurrence and development of diverse diseases. However, the specific mechanism that determines the duality of m6A and the regulatory mechanism of m6A on core genes remain unclear. Here, we summarize the latest research progress on the intricate role of the same or similar function and different m6A-related enzymes in cancers.

### The intricate role of different m6A-related enzymes in cancers

**Figure 2:**
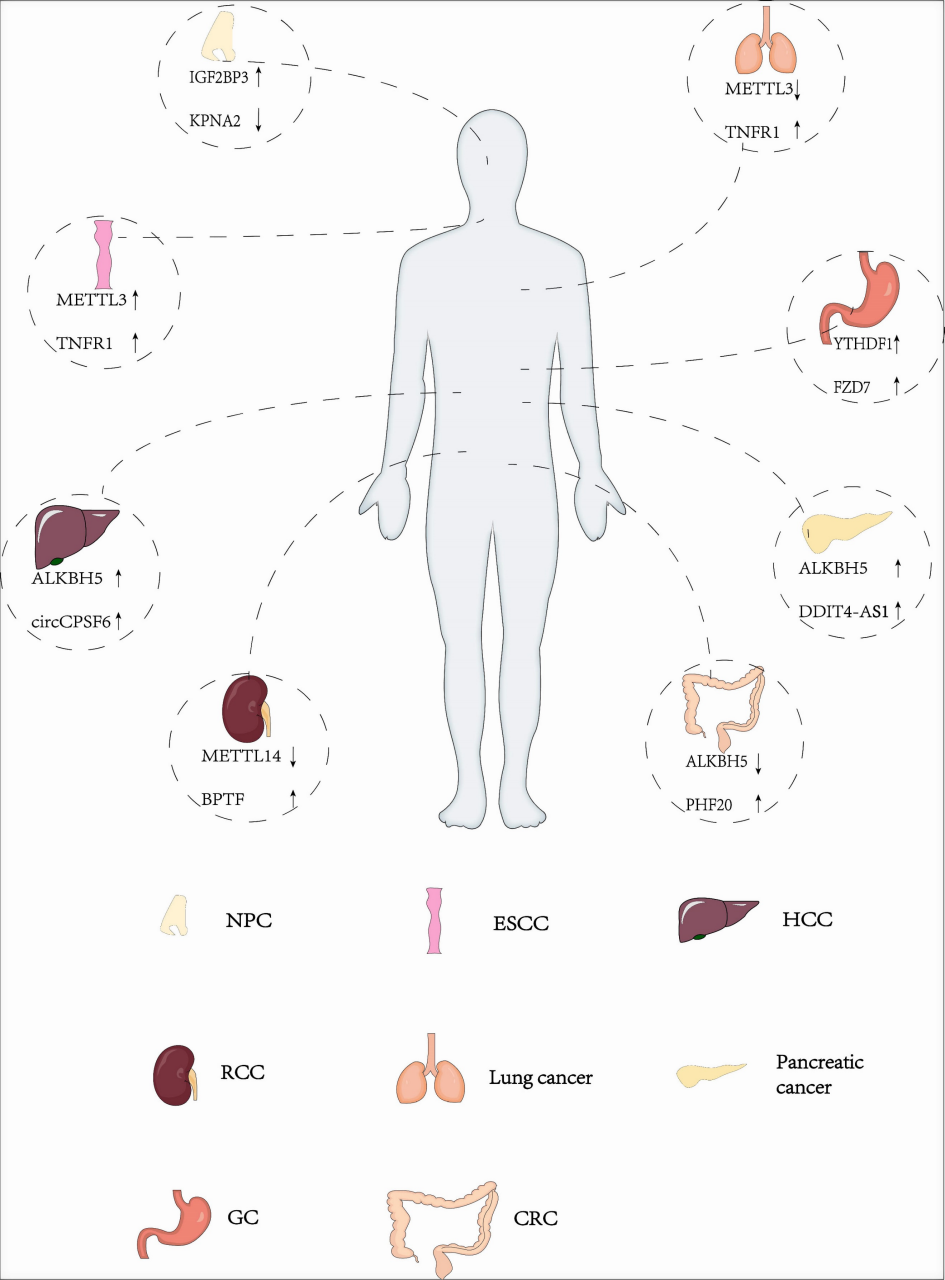
The intricate role of m6A modification in different cancers.

M6A methylation has been shown to play an important role in many cancer types, such as head and neck cancer 46, acute myeloid leukemia (AML) 47, glioblastoma (GBM) 48, nasopharyngeal carcinoma (NPC)^49^, breast cancer 50, lung cancer 51, gastric cancer 52, pancreatic cancer 53, bladder cancer 54, hepatocellular cancer (HCC) 55, colorectal cancer (CRC)^56^, endometrial cancer 57 etc (Fig.2). However, the role of m6A in different tumors is dynamic and contradictory.

WTAP can promote the progression and metastasis of NPC by increasing the stability of lncRNA DIAPH1-AS1 58. METTL3 plays oncogenic roles in esophageal squamous cell carcinoma (ESCC) by decreasing the expression of APC, a tumor suppressor gene 59. In non-small cell lung cancer (NSCLC), METTL3 induces drug resistance and metastasis through the m6A-MALAT1-YAP axis 60. Similarly, in another common type of lung adenocarcinoma (LUAD), METTL3 increases the stability of lncRNA LCAT3, leading to the binding of FUBP1, activating the oncogenic molecule c-MYC, and promoting tumor proliferation, invasion, and metastasis 61. In addition, METTL3 can promote tumor development in LUAD by stimulating ENO1 translation mediated by YTHDF1 62. Studies also revealed the critical part that m6A modification plays in drug resistance 63, 64. Recent studies have shown that m6A modification is important for immunoregulation. METTL3 depletion inhibits PD-L1 expression in an m6A-IGF2BP3-dependent manner, which in turn enhances antitumor immunity in breast cancer 65.

In GBM, the SPI1-induced downregulation of FTO promotes tumor progression by regulating pri-miR-10a processing in an m6A-dependent manner 66. In contrast, for NPM1-mutated AML, which accounts for approximately one-third of AML cases, FTO is aberrantly overexpressed and serves as a carcinogen by promoting the cell cycle and inhibiting apoptosis 67. Similarly, ALKBH5 promotes tumor growth and metastasis through the TRAF1-mediated activation of the NF-κB and MAPK signaling pathways in multiple myeloma 68.

To better understand the role of m6A in various diseases, it is important to examine methylated reading proteins that also play an integral role in tumor progression. YTHDF2 positively correlates with the grade and low prognosis of gliomas. YTHDF2 can activate NF-κB by accelerating the degradation of UBXN1 mRNA, thereby promoting glioma growth, proliferation, and metastasis 69. In addition, YTHDF1 promotes tumor progression in CRC by promoting ARHGEF2 translation and RhoA signaling 70. In AML, the m6A reader IGF2BP3 is associated with low prognosis and can accelerate the occurrence and development of tumors by increasing the stability of RCC2 71.

In conclusion, m6A plays an important role in different tumors. However, the level of m6A in different tumors is bidirectional, both pro-cancer and anti-cancer, and the specific mechanism needs to be further explored.

### The contradictory role of m6A-related enzymes with the same or similar functions in cancers

Methylation-related enzymes with opposite effects may have contradictory expression, even in the same tumor. In gastric cancer, ALKBH5 is expressed at a low level and is strongly associated with clinical tumor distal metastasis and lymph node metastasis, while the silencing of ALKBH5 promotes tumor invasion and metastasis 72. In a study of gastric cancer, METTL3 was surprisingly under-expressed in the opposite direction and the overexpression of METTL3 inhibited gastric cancer progression through the methylation modification of circORC5 73. In pancreatic cancer, ALKBH5 plays a pro-oncogenic role and ALKBH5 overexpression can promote tumor progression by downregulating potassium two-pore domain channel subfamily K member 15 and WISP2 antisense RNA 1 (KCNK15-AS1), an oncogene, to promote tumor growth invasion and metastasis 74. Conversely, METTL3, a methyltransferase, plays a pro-oncogenic role in pancreatic cancer. METTL3 accelerates the growth, invasion, and metastasis of pancreatic cancer by reducing SMS expression in an m6A-dependent manner 75.

Similarly, even m6A-related enzymes, which have the same roles in the same tumor, exhibit paradoxical roles. METTL14 is under-expressed in CRC and is associated with poor prognosis in patients. METTL14 inhibits tumor cell proliferation by abolishing the m6A level of XIST and augmenting XIST expression mediated by YTHDF2 76. In another CRC study, METTL3 shows higher expression in CRC tissues than in normal tissues, and the overexpression of METTL3 promotes tumor progression by regulating the m6A-CRB3-Hippo axis 77. METTL3 induces GLUT1 translation to promote glucose uptake and lactate production, leading to the activation of mTROC1 signaling, thereby promoting tumor progression 78. In summary, it has been suggested that m6A plays conflicting roles not only in different tumors, but also in the same tumor. The specific reasons for this may include the heterogeneity of cell and tissue samples as well as the amount of m6A that varies dynamically in tumors. The same m6A-related enzymes may have other unknown potential roles. Different m6A-related proteins that perform the same function may have different unknown roles apart from their role in methylation, which can lead to opposite outcomes. The type of molecule regulated by m6A (pro-cancer or anti-cancer) may differ. The recognition of different reading proteins of m6A and the type of disease to a greater or lesser extent are also variable. The specific mechanisms also need to be further investigated.

## The combination of m6A and PCD in cancers

**Figure 3:**
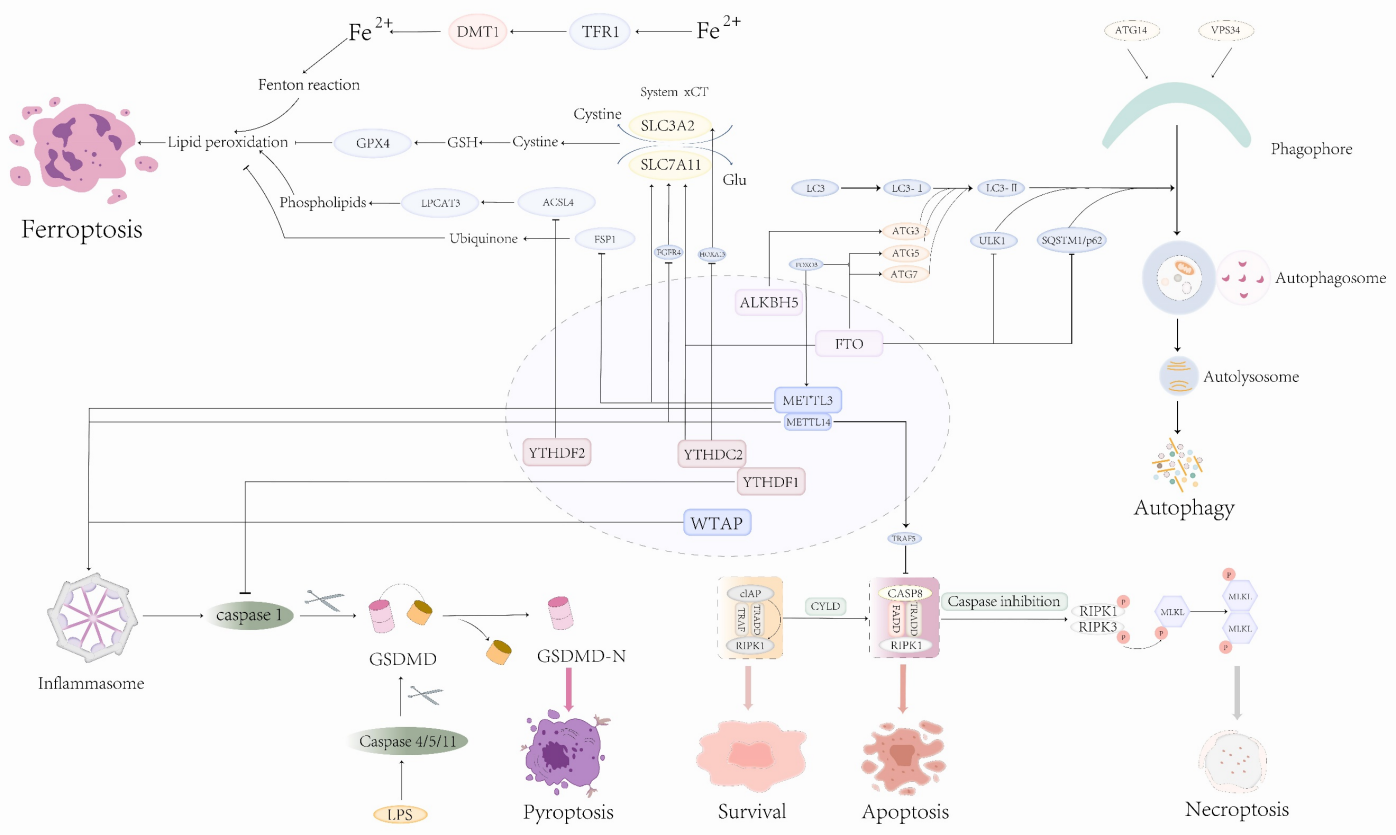
Role of m6A modification in mediating different types of PCD.

Here, we mainly discuss the connection between m6A and cell apoptosis, autophagy, pyroptosis, and ferroptosis, as well as necroptosis. M6A is controlled by regulatory factors (writers and erasers) and recognition factors (readers) to mediate downstream targets to regulate PCD (Fig. 3). It is certain that the connection of m6A and PCD pathways will provide new insights into the management of related diseases.

### M6A and apoptosis

**Figure 4:**
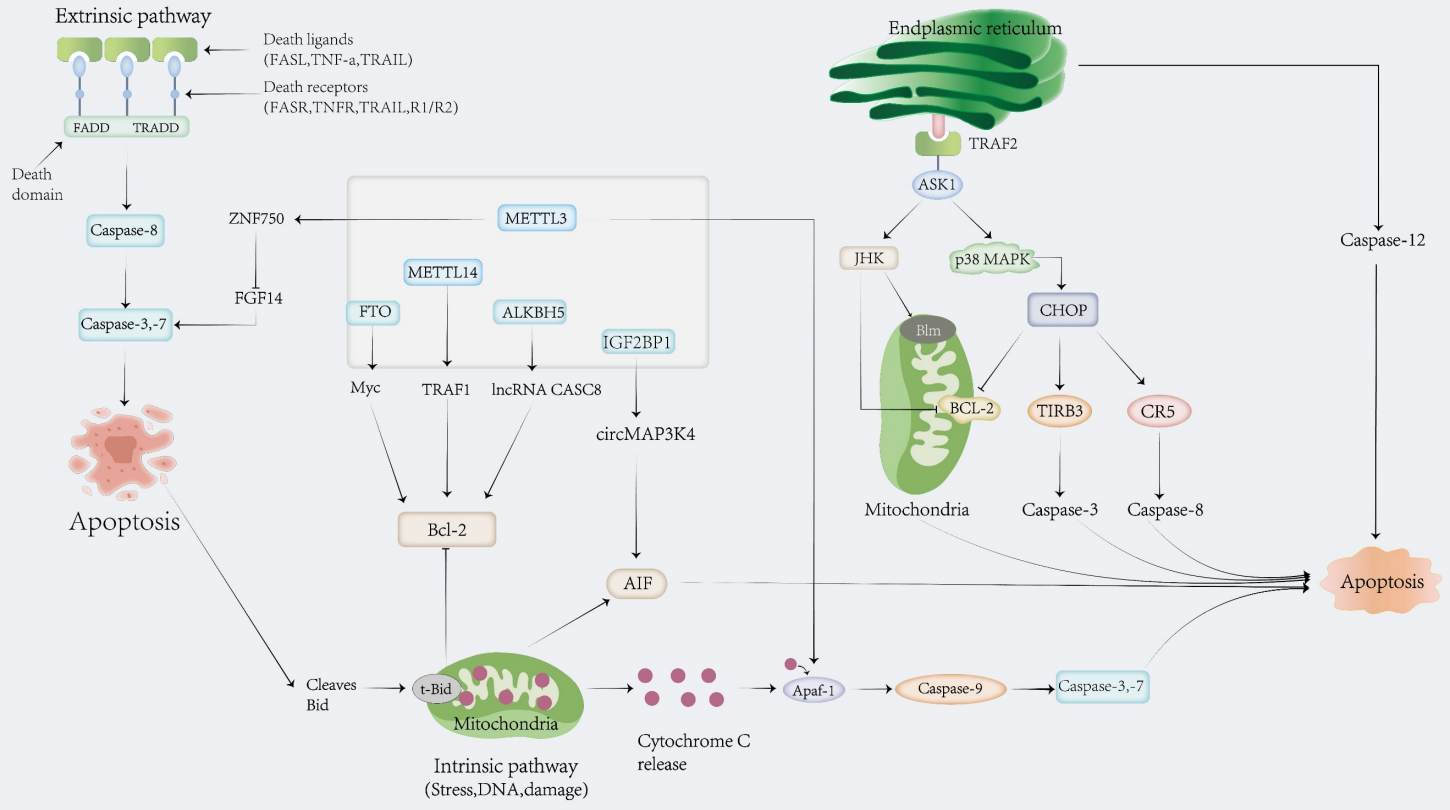
Role of m6A modification in mediating apoptosis.

Programmed death refers to physiological death, which is a predetermined and tightly programmed cellular and molecular biological process in ontogeny. As one of the modes of programmed death, the apoptosis-related signaling pathway is classified into three types: classic mitochondria, endoplasmic reticulum, and exogenous death receptor pathways 79. Apoptosis-related molecular types include the Bcl-2 subfamily and the caspase family 80, 81. Since METTL3 depletion was reported to induce apoptosis and decrease AML development in 2017 82, an increasing number of studies have revealed that apoptosis is regulated by m6A, which plays a crucial role in the occurrence and development of cancer by promoting or suppressing apoptosis (Fig.4).

In Alzheimer's disease, low levels of METTL3 lead to memory loss by promoting neuronal apoptosis with extensive synaptic loss, neuronal death, and multiple AD-related alterations, including oxidative stress and aberrant cell cycle events 83. The M6A-mediated upregulation of circMDK promotes cancer progression and apoptosis via the miR-346/874-3p-ATG16L1 axis 84. In the progression of osteosarcoma, the expression of ALKBH5 is low and its overexpression inhibits STAT3 activity to reduce cell proliferation and apoptosis in an m6A-YTHDF2-dependent manner 85. In contrast, ALKBH5 is upregulated in myeloma, and its inhibition represses the myeloma cell proliferation, invasion, and migration ability, while it promotes apoptosis 86.

Increasingly, studies have also demonstrated that the m6A-apoptosis axis plays a crucial role in the tumor microenvironment. The inhibition of IGF2BP1, as a crucial m6A reader protein, exerts a tumor suppressor effect in HCC by inducing apoptosis and subsequently activating immune cell infiltration as well as blocking PD-L1 expression to regulate the tumor immune microenvironment 87. COL10A1 secreted by cancer-associated fibroblasts (CAFs) and upregulated by METTL3, facilitates cell proliferation and represses apoptosis-induced oxidative stress in LUSC 88.

Apparently, m6A-apoptosis axis plays an indispensable role when it comes to addressing the problem of drug resistance in cancer. In a study of sunitinib resistance in renal cell carcinoma, TRAF1 increases significantly in sunitinib-resistant cells, while the TRAF1 overexpression promotes sunitinib resistance by modulating apoptosis in a METTL14-dependent manner 89. In gastric cancers, lncRNA ABL is significantly elevated, while the ABL overexpression inhibits GC cell apoptosis and enhances multidrug resistance. Mechanistically, ABL is stabilized by METTL3-mediated m6A modification and subsequently binds to APAF1 to block the apoptosome assembly and caspase-9/3 activation, thereby leading to increased sensitivity to chemotherapy 90. For chemoresistance in ESCC, the highly overexpressed ALKBH5-induced lncRNA CASC8 activate the Bcl2/Caspase3 pathway to decrease the cisplatin sensitivity of ESCC and promote tumor development 91.

### M6A and autophagy

Autophagy is an endogenous defense process that relies on autophagic lysosomes that degrade their encapsulated contents to meet the metabolic needs of the cell and the renewal of some organelles, thus playing an important role in tumor development and evolution 92. It is characterized by the formation of autophagosomes (Frankelet al., 2017), autophagy-related genes (ATG), uncoordinated 51-like kinase 1 (ULK1), and transcription factor EB (TFEB), which act as important regulators of autophagy. In 2018, the first research on m6A and autophagy was conducted. FTO deficiency promotes the expression of ULK1, a key protein associated with autophagy, to delay tumor progression in an m6A-YTHDF2-dependent manner 93. Since then, valuable insights have been provided regarding the role of m6A-related autophagy in the occurrence and development of tumors.

Impaired autophagy has also been observed in the progression of osteoarthritis-synoviocytes. In terms of the mechanism, METTL3 decreases the expression of autophagy-related 7, an E-1 enzyme crucial for the formation of autophagosomes, enhances autophagic flux, and promotes cellular senescence and osteoarthritic progression 94. In the process of malignant skin transformation and tumorigenesis, FTO is upregulated and stabilized by low-level arsenic through the inhibition of p62-mediated selective autophagy 95. Similarly, FTO is also upregulated and closely related to autophagic flux in clear cell renal cell carcinoma, whereas FTO knockdown enhances autophagic flux and impairs tumor growth and metastasis 96. Methylated proteins can also regulate autophagy. YTHDF1 deficiency inhibits HCC autophagy, growth, and metastasis by promoting the translation of autophagy-related genes ATG2A and ATG14 in an m6A-dependent manner 97.

Emerging evidence indicates that autophagy regulated by m6A also influences the efficacy of immunotherapy. Melanoma tumorigenesis and anti-PD-1 resistance are promoted by m6A mRNA demethylase FTO, which is induced by metabolic starvation stress through the autophagy and NF-κB pathways 98. In addition, m6A methylation is involved in immune infiltration and autophagy in primary Sjögren's syndrome (pSS) 99. Furthermore, emerging studies have reported that the m6A-autophagy axis plays an important role in drug resistance. For gefitinib resistance in NSCLC cells, METTL3-mediated autophagy reverses this drug resistance by regulating β-elemene 100. The upregulated lncRNA ARHGAP5-AS1 is affected by autophagy, and SQSTM1 is responsible for transporting ARHGAP5-AS1 to autophagosomes in chemo-resistant gastric cancer cells 101. In addition, METTL3 improves the resistance of HCC cells to sorafenib by stabilizing FOXO3 mediated by YTHDF1 in an m6A-dependent manner, thereby inhibiting the expression of autophagy-related genes including ATG3, ATG5, ATG7, ATG12, and ATG16L1 102.

In summary, the m6A-autophagy axis seems to be involved in the initiation and progression of different cancers and plays an important role in the immune phenotype and drug resistance. M6A-autophagy could be contributing to multifarious cancer progression and potentially represent a novel therapeutic target.

### M6A and ferroptosis

Ferroptosis, a newly discovered type of programmed cell death, is an underlying therapeutic strategy for the inhibition of cancer occurrence and development 103. Since the discovery of ferroptosis in 2012 104, an increasing number of studies have demonstrated the important role of ferroptosis in various cancers. Ferroptosis has been used to describe a highly complex process that requires the coordination of a series of signals from different organelles. The organelles involved include the endoplasmic reticulum, peroxisomes, and lysosomes. Several common mechanisms of ferroptosis are related to oxidative damage and antioxidant defense. Specifically, iron accumulates first, followed by lipid peroxidation, and finally the rupture of the cytoplasmic membrane occurs 105. Ferroptosis is an iron-dependent and non-apoptotic oxidative form of cell death, whose definitive hallmark genes are related to iron accretion and lipid peroxidation. It can be regulated at different levels, particularly in epigenetics. To date, an increasing number of studies have demonstrated a potential relationship between m6A modifications and ferroptosis (Table 1).

Doxorubicin, which plays a toxic role in the heart, upregulates METTL14 and promotes cardiomyocyte ferroptosis via the KCNQ1OT1-MIR-7-5P-TFRC axis. Therefore, targeting METTL14 and ferroptosis may provide a promising strategy for controlling DOX-induced cardiac injury 106. In addition, METTL3-mediated SLC7A11, a subunit of the Xc^-^ system, enhances the ferroptotic resistance and promotes proliferation and apoptosis in LUAD 107. Similarly, m6A medicated SLC7A11 in ferroptosis has been found in hepatoblastoma 108, glioblastoma 109, thyroid cancer 110, and LUAD 111. For FSP1, an iron suppressor protein 1, another key factor in ferroptosis, miR-4443 is highly expressed in cisplatin-resistant tissue-derived exosomes in NSCLC, and miR-4443 overexpression can pass METLL3 by cisplatin treatment. This inhibits FSP1-mediated ferroptosis and promotes tumor growth 112.

In addition, m6A reading proteins have also been demonstrated to play an indispensable role in cancer. In LUAD, YTHDC2 is a ferroptosis inducer that promotes the development of cancer by targeting the SLC3A2 subunit of system Xc^-^ 111.

Ferroptosis plays an important role in drug resistance. In recalcitrant HER2-positive breast cancer, FGFR4 knockdown reduces resistance to anti-HER2 therapy by activating ferroptosis. Mechanistically, FGFR4, modified by m6A, blocks glutathione synthesis and Fe^2+^ efflux efficiency via the β-catenin/TCF4-SLC7A11/FPN1 axis, resulting in excessive ROS production and labile iron pool accumulation 113. Hypoxia-induced lncRNA-CBSLR increases chemoresistance in gastric cancer by inhibiting ferroptosis. In detail, CBSLR decreases the stability of CBS mRNA in an m6A-YTHDF2-dependent manner, leading to the polyubiquitination and degradation of ACSL4, which decreases the pro-ferroptotic phosphatidylethanolamine (PE) (18:0/20:4) and PE (18:0/22:4) content to inhibit the activation of ferroptosis 114. In NSCLC, m6A-medicated miR-4443 also enhances cisplatin resistance through the inhibition of ferroptosis 112.

Although several ferroptosis-associated lncRNAs have been analyzed for their correlation with m6A-related genes in terms of immune efficacy 115, 116, 117, few studies have investigated the specific targets or pathways of m6A and ferroptosis in immune therapy. Given that ferroptosis and m6A play critical roles in tumors, further research on m6A-modified ferroptosis in diverse cancers is needed.

### M6A and pyroptosis

Pyroptosis, a lytic form of cell death, is characterized by NLR pyrin domain containing 3 (NLRP3), apoptotic speck-like protein containing CARD (ASC), cleaved Caspase-1, Gasdermin-D (GsdmD) p30, IL-1β, and IL-18, which serve as important regulators of pyroptosis 120. Although an increasing number of studies have demonstrated the critical role of pyroptosis or m6A in different diseases, including different cancers 121, studies on the m6A-pyroptosis axis in cancer seem to be few, while most focus on ischemic diseases and some chronic diseases (Table 2). In hypoxic pulmonary hypertension, the degradation of lncRNA FENDRR mediated by YTHDC1 promotes HPAEC pyroptosis by regulating DRP1 promoter methylation 122. In slow-transit constipation, METTL3 promotes the pyroptosis of glutamic acid-induced ICCs by interacting with DGCR8 and modulating the miR-30b-5p/PIK3R2 axis in an m6A-dependent manner 123.

In terms of m6A-pyroptosis in ischemia-reperfusion injury, hypothermia protects neurons from cerebral ischemia-reperfusion injury by downregulating the secretion of the pyroptosis-related proteins NLRP3, ASC, and some pro-inflammatory factors by activating PI3K/Akt signaling via the m6A modification of PTEN mRNA 124. Meanwhile, METTL3 promotes pyroptosis in myocardial cells to exacerbate myocardial ischemia-reperfusion injury in an m6A-dependent manner 125. The m6A-pyroptosis axis seems to play an important role in ischemia-reperfusion injury, which is consistent with the results of a similar summary 126, but the regulation of m6A-pyroptosis at other sites of ischemia-reperfusion injury and more specific mechanisms remain to be explored.

Additionally, m6A-mediated pyroptosis occurs in some chronic diseases. In patients with diabetic nephropathy (DN), WTAP is highly expressed, and WTAP knockdown inhibits the m6A methylation of NLRP3 mRNA to downregulate NLRP3 inflammasome activation, which further induces cell pyroptosis and inflammation 127. Similarly, in diabetic nephropathy, the total flavones of *Abelmoschus manihot* (TFA) inhibit the pyroptosis of podocytes under high glucose conditions by regulating METTL3-dependent m6A modification and inhibiting the activation of the NLRP3 inflammasome and PTEN/PI3K/Akt signaling 128. Additionally, METTL3 expression is lower in T2DM patients. The overexpression of METTL3 alleviates high-glucose-induced apoptosis and pyroptosis in human retinal pigment epithelial (RPE) cells via the METTL3/miR-25-3p axis 129. The same mechanism is involved in diabetic cardiomyopathy. METTL3 degrades lncRNA TINCR mediated by YTHDF2, which further decreases the expression of NLRP3, a key pyroptosis-related protein, to regulate the occurrence of pyroptosis and diabetic cardiomyopathy 130. In diabetic retinopathy, circFAT1 interacts with YTHDF2, which increase the expression of LC3B to promote autophagy and inhibit pyroptosis in high glucose-induced retinal pigment epithelial (RPE) cells 131. The m6A-pyroptosis axis appears to play a profound role in the chronic complications of diabetes, but more research is needed to extend our understanding of the epigenetic regulation of pyroptosis in DCM progression. Interestingly, the m6A-pyroptosis is not restricted to epithelial cells and is involved in some immune cells. In atherosclerosis and acute coronary syndrome (ACS), IRF-1 represses circ-0029589 expression in an METTL3 dependent manner, thereby promoting macrophage pyroptosis and inflammatory responses 132. In addition, the METTL3/MALAT1/PTBP1/USP8/TAK1 axis in liver fibrosis promotes pyroptosis and macrophage M1 polarization, thereby exacerbating liver fibrosis progression 102. Although m6A-modified pyroptosis plays an important role in non-neoplastic diseases, its role in tumors requires further study.

### M6A and necroptosis

Necroptosis is a form of programmed necrosis that occurs when apoptosis is blocked by extracellular signals (death receptor-ligand binding) or intracellular triggers (microbial nucleic acids) through a series of phosphorylation events that result in the production of pore complexes on the plasma membrane by MLKL, leading to the secretion of DAMP and subsequent cellular self-destruction 136. Necroptosis is characterized by organelle swelling, cell membrane rupture, and the disintegration of the cytoplasm and nucleus, with RIPK1, RIPK3, and MLKL as the main molecules involved 137. In addition, tumor necrosis factor (TNF), Toll-like receptor (TOLLR) family members, interferon, and other mediators have been demonstrated to act as key genes in necroptosis 138.

In CRC, patients resistant to oxaliplatin have higher METTL3 expression and infiltration of M2-type macrophages. Further studies revealed that the TRAF5-mediated inhibition of necroptosis contributes to METTL3-triggered OX resistance. This finding shows the role of the m6A-necroptosis axis in OX resistance and provides a new target for patients with OX resistance in CRC 139. In addition, necroptosis-related genes, such as necroptosis-related mRNA and necroptosis-related lncRNA, are indicators of a worse prognosis and correlate with m6A gene expression and immune function, which are used to predict the prognosis and immune response in different cancers; however, the specific mechanism remains need to be further investigated 140, 141, 142, 143. Few studies have examined the role of m6A modification in necroptosis; therefore, the precise regulatory mechanisms involved are still unknown.

### Small molecular compounds targeting m6A modification in various cancers

The m6A modification has been found to act as a pivotal role in various cancers. Therefore, inhibitors and regulators targeting m6A regulators may be effective new approaches for cancer therapy. STM2457, a highly potent and selective first-in-class catalytic inhibitor of METTL3, was demonstrated to hinder the growth of AML without impacting normal hematopoiesis 144. It had also been proven to block the proliferation of intrahepatic cholangiocarcinoma in an m6A-YTHCF2-dependent manner 145. In addition to METTL3, other m6A regulators are also key targets for treating cancers with abnormal m6A levels. Rhein, competitively bounds to FTO or AlkB catalytic *in vitro*, displayed the enhancement of antiproliferative effects of atezolizumab based on breast cancer (4T1) regression 146. It also examined the inhibitory effect in breast cancer *in vitro* and *in vivo* 147. Meclofenamic acid (MA), a highly selective inhibitor of FTO, restored gefitinib sensitivity via FTO/m6A-Demethylation/c-Myc in Non-Small Cell Lung Cancer 148. It also played a protective effect in cisplatin-induced acute kidney injury 149. In glioma, MA2 (the ethyl ester form of meclofenamic acid) inhibited FTO and suppressed proliferation by increasing the effect of the chemotherapy drug temozolomide 150. MA2 could also rescue the cisplatin-induced cytotoxicity of bladder cancer cells 151. Subsequently, other FTO inhibitors, such as MO-I-500, FB23-2, R-2HG, CS1, and CS2, are also showed the antitumor effect on diverse cancers, including Alzheimer's disease (AD) 152, nasopharyngeal carcinoma 153, Breast Cancer 154, AML 155, 156, 157, cholangiocarcinoma 158, renal cell carcinoma 96. Furthermore, IGF2BP1 acts as the post-transcriptional super-enhancer of E2F-driven gene expression in cancer. The small molecule, BTYNB, could disrupt this enhancer function by impairing the IGF2BP1-RNA association. It also showed the inhibitory potency in the treatment of solid cancers 159. In conclusion, although the small molecular compounds targeting m6A modification for clinical application is still in the initial phase, it is expected that drugs targeting m6A modification will be improved and developed and finally be used for clinical treatment over the next few years with more understanding of the function and mechanism of m6A in cancer.

### Potential therapeutic applications of m6A-modified PCD in cancers

Increasing evidence has gradually proven that an anti-tumor strategy based on PCD and m6A may solve some existing problems in anti-cancer therapies (Table 3). In chemotherapy efficacy and resistance, diverse types of regulators of PCD and m6A molecules show efficacy in the chemoresistance of tumor cells 160, 161. The combination of m6A and PCD also improves anti-cancer efficacy. In the m6A-apoptosis axis, WTAP knockdown facilitates cell apoptosis and inhibits cisplatin resistance in nasal-type natural killer/T-cell lymphoma 162. Conversely, FTO enhances chemoresistance in CRC through SIVA1-mediated apoptosis via a YTHDF2-dependent mechanism 163. In terms of m6A-autophagy, METTL3-mediated autophagy reverses gefitinib resistance in NSCLC cells by β-elemene 100. In seminomas, METTL3 regulates autophagy and sensitivity to cisplatin by targeting ATG5 164. 5-Azacytidine, a methyltransferase inhibitor and anticancer drug, stimulates an autophagic response to sensitize cancer cells to drug responsiveness during hydrogen peroxide-induced oxidative stress in insulinoma β-TC-6 cells 165. Regarding the m6A-ferroptosis axis, METTL14-modified FGFR4 increases anti-HER2 resistance by inhibiting ferroptosis mediated by the β-catenin/TCF4-SLC7A11/FPN1 axis in recalcitrant HER2-positive breast cancer 113. The low level of hypoxia-inducible lncRNA-CBSLR manifests a worse clinical outcome and a poorer response to chemotherapy, and regulates ferroptosis and chemoresistance through m6A-YTHDF2-dependent modulation 114. In terms of the m6A-pyroptosis axis, recent research indicated that the lower expression levels of DFNA5/GSDME in most tumor cells than in normal cells is attributed to the methylation of mRNA, thus making it difficult to activate pyroptosis to increase the sensitivity of chemotherapeutic drugs in most tumor cells. Therefore, appropriate chemotherapeutic drugs can be selected based on the expression levels of DFNA5/GSDM, in order to increase their effects 166. In immune therapy, ALKBH5-dependent HMGB1 expression decreases hepatocyte apoptosis and mediates the STING-IFN regulatory factor 3 innate immune response in radiation-induced liver diseases 167. FTO promotes melanoma processing and anti-PD-1 resistance, and suggests the potential of the combination of FTO inhibition with anti-PD-1 blockade in resistance to immunotherapy 98.

In conclusion, m6A-PCD may play a critical role in drug efficacy and resistance, including chemotherapy resistance, immune efficacy, and drug side effects in m6A-apoptosis. An increasing number of studies on m6A-PCD have focused on m6A autophagy and m6A-apoptosis. However, few studies have investigated the therapeutic application of m6A-modified pyroptosis and necroptosis, and further studies are needed.

## Summary

Emerging studies have shown that m6A modification affects apoptosis and autophagy, thereby influencing the development of diverse cancers. However, few studies have examined the effects of m6A modification on ferroptosis, pyroptosis, and necroptosis in cancer. M6A-pyroptosis mainly focuses on non-neoplastic diseases, while m6A-necroptosis has been rarely studied in cancer. In brief, these studies revealed that m6A plays a remarkably important role in PCD and tumor development. However, the influence of m6A modification on PCD remains largely unclear, similar to the contradictory role of m6A modification in diverse cancers. For instance, METTL3 acts as an apoptotic driver in LUAD 173 whereas WTAP serves as a suppressor in endometrial cancer 174. In addition, m6A modification serves as an autophagy driver in NSCLC 100 whereas METTL3 functions as a suppressor in HCC 175. Whether PCD regulated by m6A exerts a promotive or inhibitive effect may primarily depend on the level of m6A (the dynamic balance between writers and erasers), the different readers, the variety (mRNA or ncRNA), the function of target genes, and different diseases.

More importantly, the mechanism of methylation modification of all m6A-related proteins is not the same, the diverse biological processes which be affected by m6A modification is not specificity, the target genes may be regulated by diverse readers, and the m6A-related proteins identified thus far may have more than one role beyond methylation, which may lead to diametrically opposed roles in disease.

Interestingly, in addition to m6A, which regulates PCD, it also affects the m6A levels. The release of neutrophil extracellular traps (NETs) activates ferroptosis depending on the METTL3-induced m6A modification of GPX4 in sepsis-associated acute lung injury 176. Meanwhile, NET-activated METTL3 leads to abnormal autophagy in sepsis-associated acute lung injury 177.

Moreover, except for the regulation of m6A in a single type of PCD, m6A indirectly regulates one type of PCD through another type of PCD. For example, in hepatic stellate cells, m6A-modified BECN1 promotes the activation of autophagy, thus inducing ferroptosis 178. M6A-medicated PCD may be an indirect way to influence the activation of other PCD signals. It is believed that further studies on PCD interactions will provide an explanation. Further studies are needed to explore the mutual link between m6A modification and PCD in different diseases.

In addition, m6A modification could also happen on TAM, especially in immune cells, to indirectly effect tumor development. The balance between cancer cells and TAM may account for the contradictory role of m6A and PCD. Studies have shown that RNA modification is involved in the development, differentiation, activation, migration, polarization and other biological processes of immune cells, thus regulating immune response and participating in the occurrence of some immune-related diseases 179. Such as m6A methyltransferase in TAMs promotes CD8+ T cell dysfunction and tumor progression 180. It also revealed that the immune cell can also occur PCD to influence the development of cancers. But the specific mechanisms that tie them together still require further study. Moreover, except for m6A, other chemical modifications in DNA, RNA, and protein are also irreplaceable, such as DNA methylation, m1A, m5C, ubiquitylation, Phosphorylation, lactation, glycosylation modification, and so on. Whether these epigenetic modifications play an indispensable role as m6A in PCD? Are the other types of PCD, such as immunogenic cell death and cuproptosis, also regulated by m6A or other epigenetic modifications? These questions remain needed to be further explored.

In general, m6A-associated targets would provide a new direction for clinical diagnosis, treatment, prognosis, and therapy resistance in cancer. In general, investigating the intricate relationship between m6A and PCD could improve our understanding of how certain diseases develop and lead to the development of new treatments.

## Author contributions

Q.T., M.Y. and J.C. organized the whole topic. J.C., M.Y., J.B. and C.H. drafted the manuscript. F.L., D.G. and P.Y. revised the manuscript. All authors read and approved the final manuscript.

## Figures and Tables

**Table 1:** M6A-ferroptosis axis in diverse cancers.

Disease models	m6a regulation	Type of PCD	Biofunction	Reference
Glioblastoma	METTL3	ferroptosis	RNA binding protein NKAP promotes SLC7A11 mRNA splicing in an m6A-dependent manner.	(109)
Thyroid Cancer	FTO	ferroptosis	FTO can inhibit the development of Thyroid cancer by downregulating SLC7A11 in m6A independently.	(110)
	ALKBH5	ferroptosis	ALKBH5 inhibits thyroid cancer progression by promoting ferroptosis through TIAM1-Nrf2/HO-1 axis.	(118)
Non-Small Cell Lung Carcinoma	METTL3	ferroptosis	miR-4443 in cisplatin-resistant NSCLC tumor tissue-derived exosomes regulated the expression of FSP1 in an m6A manner via METLL3.	(112)
Lung Adenocarcinoma.	YTHDC2	ferroptosis	YTHDC2 suppressed SLC3A2 via inhibiting HOXA13 in an m6A-indirect manner.	(111)
Breast Cancer	METTL14	ferroptosis	METTL14 regulated FGFR4 diminishes glutathione synthesis and Fe2+ efflux efficiency via the β-catenin/TCF4-SLC7A11/FPN1 axis.	(113)
Gastric Cancer	YTHDF2	ferroptosis	lncRNA-CBSLR interacted with YTHDF2 to form a CBSLR/YTHDF2/CBS signaling axis to reduce the expression of ACSL4.	(114)
Hepatocellular Carcinoma	IGF2BP3	ferroptosis	IGF2BP3-NRF2 axis regulates ferroptosis in hepatocellular carcinoma.	(119)
Hepatoblastoma	METTL3	ferroptosis	METTL3-mediated SLC7A11 m6A modification enhances HB ferroptosis resistance in an IGF2BP1 dependent manner.	(108)

**Table 2:** M6A-pyrpotosis axis in no-cancer diseases.

Hypoxic pulmonary hypertension	YTHDC1	pyroptosis	YTHDC1-induced decay of lncRNA FENDRR promotes HPAEC pyroptosis by regulating DRP1 promoter methylation.	(122)
Slow transit constipation	METTL3	pyroptosis	METTL3 contributes to slow transit constipation by regulating miR-30b-5p/PIK3R2/Akt/mTOR signaling cascade through interacting with DGCR8.	(123)
Sepsis	YTHDF1	pyroptosis	YTHDF1 alleviates sepsis by upregulating WWP1 to induce NLRP3 ubiquitination and inhibit caspase-1-dependent pyroptosis.	(133)
Liver fibrosis	METTL3	pyroptosis	The METTL3/MALAT1/PTBP1/USP8/TAK1 axis promotes pyroptosis and M1 polarization of macrophages and contributes to liver fibrosis.	(134)
Atherosclerosis (AS) and acute coronary syndrome (ACS)	METTL3	pyroptosis	IRF-1 can repress circ-0029589 expression in a METTL3-dependent manner, thereby promoting macrophage pyroptosis and inflammatory responses	(132)
Myocardial Ischemia-Reperfusion Injury	METTL3	pyroptosis	METTL3 promotes DGCR8 binding to pri-miR-143-3p in an m6A dependent manner, thus enhancing miR-143-3p expression to inhibit PRKCE transcription and further aggravating cardiomyocyte pyroptosis and MI/R injury.	(125)
Cerebral ischemia/reperfusion (I/R) injury	m6A	pyroptosis	Hypothermia protects neurons against ischemia/reperfusion-induced pyroptosis via m6A-mediated activation of PTEN and the PI3K/Akt/GSK-3β signaling pathway.	(124)
Diabetic nephropathy	WTAP	pyroptosis	WTAP promotes the expression of NLRP3 in a IGFBP2 dependent manner to upregulate NLRP3 inflammasome activation, which further induces cell pyroptosis and inflammation.	(127)
Diabetic nephropathy	METTL3	pyroptosis	TFA can ameliorate pyroptosis by regulating the expression of METTL3 and regulating NLRP3-inflammasome activation and PTEN/PI3K/Akt signaling.	(128)
Diabetic cardiomyopathy (DCM)	METTL14	pyroptosis	METTL14 suppresses pyroptosis and DCM via downregulating lncRNA TINCR, which further decreases the expression of key pyroptosis-related protein, NLRP3.	(135)
Diabetic retinopathy	YTHDF2	pyroptosis	CircFAT1 interact with YTHDF2 to increase the expression of LC3B, thus promoting autophagy and inhibiting pyroptosis of RPE cells induced by HG.	(131)

**Table 3:** The potential clinical application of m6A-modified PCD

M6A associated molecules	Target gene	Type of PCD	Disease models	Clinical application	Biofunction	Reference
IGF2BP1	PDL1	apoptosis	hepatocellular carcinoma	immunotherapy	The inhibition of IGF2BP1 inhibits the development of hepatocellular carcinoma through activating immune cells infiltration and blocking PD-L1 expression to regulate the tumor immune microenvironment.	(87)
METTL3	COL10A1	apoptosis	LUSC	immunotherapy	COL10A1 secreted by Cancer-associated fibroblasts (CAFs), upregulated by METTL3, can promote LUSC cell proliferation and repress apoptosis-induced oxidative stress.	(88)
ALKBH5	HMGB1	apoptosis	radiation-induced liver diseases (RILD)	immunotherapy	ALKBH5-modicated HMGB1 expression mediates STING-interferon regulatory factor 3 innate immune response.	(167)
FTO	PDK1	apoptosis	glioblastoma multiforme	temozolomide chemoresistance	Long noncoding RNA just proximal to X-inactive specific transcript promotes stability of PDK1 mRNA in an m6A-dependent manner.	(168)
METTL14	TRAF1	apoptosis	renal cell carcinoma	sunitinib resistance	TRAF1 overexpression can promote sunitinib resistance by modulating apoptotic in a METTL14-dependent manner.	(89)
METTL3	microRNA-221-3p	apoptosis	breast cancer	adriamycin resistance	METTL3 accelerates pri-microRNA-221-3p maturation in a m6A-dependent manner.	(169)
ALKBH5	LncRNA CASC8	apoptosis	esophageal squamous cells	cisplatin resistance	LncRNA CASC8 overexpression can activate the Bcl2/caspase3 pathway to decrease the cisplatin sensitivity of esophageal squamous cells.	(91)
METTL3	TRIM11	apoptosis	nasopharyngeal carcinoma	chemoresistance	METTL3-medicated TRIM11 promoted Daple ubiquitin-mediated degradation to upregulate β-catenin expression, thus inducing ABCC9 expression.	(170)
METTL3	lncRNA SNHG17	apoptosis	lung adenocarcinoma	gefitinib resistance	METTL3-induced lncRNA SNHG17 reduces the expression of LATS2.	(171)
METTL3	miR-146a-5p	apoptosis	bladder cancer	Melittin resistance	METTL3-guided m6A modification can accelerate the pri-miR-146 maturation.	(172)
FTO	PD-1	autophagy	melanoma	immunotherapy	FTO can increase the anti- PD-1 resistance of melanoma through the autophagy and NF-κB pathway.	(98)
METTL3	β-elemene	autophagy	non-small cell lung cancer	gefitinib resistance	METTL3-mediated autophagy can reverse this gefitinib resistance by the regulation of β-elemene.	(100)
METTL3	FOXO3	autophagy	HCC	sorafenib resistance	METTL3 can improve the sorafenib resistance of HCC cells through stabilizing forkhead box class O3 (FOXO3) mediated by YTHDF1 and inhibiting the occurrence of autophagy.	(102)
METTL14	FGFR4	ferroptosis	breast cancer	anti-HER2 therapy	METTL14 medicated FGFR4 can reduce the resistance to anti-HER2 therapy through the activation of ferroptosis by blocking glutathione synthesis and Fe2+ efflux efficiency.	(113)
METLL3	miR-4443	ferroptosis	non-small cell lung cancer	cisplatin resistance	METLL3 medicated miR-4443 can regulate the expression of FSP1 to increase the resistance to cisplatin and promote tumor growth.	(112)

## Abbreviations

m6A: N6-methyladenosine methylation
PCD: programmed cell death
ncRNA: non-coding RNA
METTL3/14: methyltransferase-like protein 3/14
METTL5/16: methyltransferase-like protein 5/16
WTAP: Wilms tumor-associated protein
VIRMA: vir-like m6A methyltransferase associated
RBM15RNA: binding motif protein 15
MTC: methyltransferase complex
SAM: S-adenosylmethionine
ZC3H13: zinc finger CCCH domain-containing protein 13
FTO: obesity-associated protein
ALKBH3/5: α-ketoglutarate-dependent dioxygenase alkB family member 3/5
YTH: domain-containing family protein (YTHDF1-3 and YTHDC1-2)
HNRNP: heterogeneous nuclear ribonucleoprotein
eIF3: eukaryotic translation initiation factor 3
IGF2BP1/2/3: insulin-like growth factor-2 mRNA-binding proteins 1/2/3
AML: acute myeloid leukemia
GBM: glioma
NPC: nasopharyngeal carcinoma
HCC: hepatocellular carcinoma
CRC: colorectal cancer
NSCLC: non-cellular lung cancer
ATGs: autophagy-related homolog
BC: breast cancer
Bcl-2: BCL2 apoptosis regulator
(KCNK15-AS1): potassium two pore domain channel subfamily K member 15 and WISP2 antisense RNA 1
GLUT1: glucose transporter 1
CAFs: cancer-associated fibroblasts
ATG: autophagy-related genes
ULK1: uncoordinated 51-like kinase 1
TFEB: transcription factor EB
pSS: Sjögren's syndrome
FOXO3: forkhead box class O3
NLRP3: NLR pyrin domain containing 3
ASC: apoptotic speck-like protein containing CARD
GsdmD: gasdermin-D
DN: diabetic nephropathy
TFA: total flavones of *Abelmoschus manihot*
RPE: retinal pigment epithelial
HG: high glucose
AS: atherosclerosis
ACS: acute coronary syndrome
NKTCL: nasal-type natural killer/T-cell lymphoma
5-azaC: 5-azacytidine
RILD: radiation-induced liver diseases
NETs: neutrophil extracellular traps
GPX4: glutathione peroxidase 4
lncRNAs: long non-coding RNAs
LUAD: lung adenocarcinoma
